# Independent and joint associations of body mass index, waist circumference, waist-height ratio and their changes with risks of hyperuricemia in middle-aged and older Chinese individuals: a population-based nationwide cohort study

**DOI:** 10.1186/s12986-021-00590-z

**Published:** 2021-06-13

**Authors:** Zonglei Zhou, Kunpeng Li, Xianzhi Li, Rongsheng Luan, Ruzhen Zhou

**Affiliations:** 1 West China School of Public Health and West China Fourth Hospital, Sichuan University, Chengdu, 610041 China; 2 Department of Neurorehabilitation, Shanghai Second Rehabilitation Hospital, Shanghai, 200441 China; 3 Department of Colorectal Surgery, Changhai Hospital of Shanghai, Shanghai, 200433 China

**Keywords:** Body mass index, Waist circumference, Waist-height ratio, Hyperuricemia

## Abstract

**Background:**

Previous reports regarding the predictive power of adiposity indices remain inconsistent, and longitudinal studies on this top are limited. The associations of hyperuricemia risk with changes in obesity status, as well as the joint effects of baseline adiposity indices and body adiposity change on hyperuricemia risk are not fully elucidated. This study aimed to explore the independent and joint associations of baseline adiposity indicators and body adiposity change with hyperuricemia risk among middle-aged and older population in China.

**Methods:**

A total of 2895 participants aged ≥ 45 years from the baseline survey of the China Health and Retirement Longitudinal Study were followed up for 4 years. Anthropometric parameters (weight, height, and waist circumference) and serum uric acid were obtained using standard devices. Adjusted odds ratio and 95% confidential interval were calculated to estimate the associations between predictor variables and hyperuricemia risk using multivariate logistic regression.

**Results:**

Of the 2895 participants, 293 (10.12%) cases of hyperuricemia were identified. Increased baseline body mass index (BMI), waist circumference, and waist-height ratio (WHtR) were significantly associated with higher risks of hyperuricemia. A slightly greater but non-significant area under the curve value was observed for waist circumference (0.622) than for BMI (0.611) and WHtR (0.614) (*P* = 0.447). Compared to subjects with stable adiposity status, participants with weight loss of ≥ 4 kg or waist circumference loss of ≥ 6 cm had a 56% or 55% lower risk of hyperuricemia, and those with weight gain of > 4 kg had a 1.62-fold higher risk of hyperuricemia. Compared to those without obesity, participants with incident or persistent obesity were more likely to develop hyperuricemia. Additionally, regardless of stable or increased weight/waist circumference during follow-up, individuals with obesity at baseline had a higher risk of incident hyperuricemia.

**Conclusion:**

This study demonstrates that BMI, waist circumference, and WHtR equally predict the development of hyperuricemia, and weight loss and waist circumference reduction are favorable in preventing hyperuricemia.

## Background

Hyperuricemia, a metabolic disease due to disorder of purine metabolism, affects 13.3% of the general population in China [1], 21.3% in the U.S. [2], and 25.8% in Japan [3]. Both genetic and environmental factors may account for the development and progression of hyperuricemia [4], nevertheless, the underlying pathological mechanisms remain incompletely elucidated. Furthermore, long-term treatment of hyperuricemia carries both risks and costs. Hence, a better understanding of risk factors for hyperuricemia and early initiation of prevention are crucial to reduce the disease burden.

Uric acid, the major end product of purine metabolism, is primarily excreted by the kidney. Accordingly, the imbalance between production and excretion of uric acid contributes to hyperuricemia [5]. Growing evidence has shown that higher body mass index (BMI) increases the risks of renal impairment, leading to a decrease in uric acid clearance [6, 7]. Furthermore, higher BMI can disrupt the glucose and insulin metabolism [8], which may subsequently impair the renal handing of uric acid and alter SUA concentration [9]. In addition, visceral fat accumulation along with increased body weight promotes the synthesis of uric acid [10]. Notably, adipose tissue has also been reported to be a site of hypoxanthine secretion and uric acid production [5, 11]. Therefore, interventions aiming to reduce BMI and waist-height ratio (WHtR) might be effective in lowering the incidence of hyperuricemia.

Previous studies have found a positive association of hyperuricemia with BMI, waist circumference, and WHtR [12–14]. However, which adiposity indicator may better predict incident hyperuricemia remains inconsistent [15, 16]. Additionally, most of these studies are cross-sectional, and there is a lack of longitudinal studies with a nationally representative sample to validate these findings in the Chinese population. Although previous reports showed a significant association of serum uric acid (SUA) concentration with changes in weight and waist circumference [17], evidence regarding the impacts of changes in obesity status on hyperuricemia risk is limited and not fully elucidated. Furthermore, no study has explored the joint effects of BMI, waist circumference, and changes in body weight or waist circumference on hyperuricemia risk.

Therefore, the present study aims to investigate the independent and joint effects of BMI, waist circumference, WHtR, and their changes on risks of hyperuricemia in middle-aged and older Chinese population.

## Methods

### Study population and procedure

The data used in this study are from the China Health and Retirement Longitudinal Study (CHARLS), a nationally representative longitudinal survey of the Chinese population aged ≥ 45 years. A detailed description is available in a previous study [18]. Briefly, participants were randomly recruited through a four-stage probability sampling approach, which involved the selection of county-level units, primary sampling units within each selected county (administrative villages in rural areas and neighborhoods in urban areas), household units, and individual units. The baseline survey was carried out between 2011 and 2012 (wave 1), three subsequent follow-ups were conducted in 2013 (wave 2), 2015 (wave 3), and 2018 (wave 4), respectively. In the present study, information collected at wave 1 and wave 3 was used due to the availability of laboratory data on SUA. The inclusion criteria included: (a) aged ≥ 45 years; (b) without hyperuricemia, kidney diseases, or undertaking chemotherapy for malignancies; (c) no missing data on BMI, waist circumference, WHtR; (d) completed the questionnaire and blood sample collection; (e) successfully followed up to wave 3. The CHARLS was approved by the Institutional Review Board of Peking University (IRB00001052-11015, IRB00001052-11014), and written informed consent was obtained from all participants.

### Data collection

Face-to-face interviews were administrated to collect data on demographic information (age, gender, residence, marital status, and education), smoking history, and drinking status. The information on comorbidities (hypertension, dyslipidemia, diabetes mellitus, and stroke) was also obtained by self-reports. Depression was evaluated using the Center for Epidemiologic Studies Depression Scale (CES-D), a 10-item questionnaire assessing the depressive feelings and behaviors in the last week [19]. The CES-D scale exhibited a good consistency in the present study, with Cronbach’s coefficient of 0.80. A total score of ≥ 10 was used to identify depression [20].

Weight, height, and waist circumference were objectively measured by trained investigators using standard devices, and were recorded to one decimal place (0.1 kg or 0.1 cm). BMI was calculated as weight in kilograms divided by height in meters squared, and was divided into four groups: underweight (< 18.5 kg/m^2^), normal weight (18.5–23.9 kg/m^2^), overweight (24–27.9 kg/m^2^), and obesity (≥ 28 kg/m^2^) [21]. WHtR was calculated as waist circumference in centimeters divided by height in centimeters [22]. General obesity was defined as a BMI value of ≥ 28 kg/m^2^. Abdominal obesity was determined if meeting one of the following criteria: (a) a waist circumference value of ≥ 90 cm for men, and a value of ≥ 85 cm for women [23]; (b) a WHtR value of ≥ 0.5 [24]. Change in weight or waist circumference was calculated as weight or waist circumference at follow-up minus the corresponding values at baseline. Weight change was divided into five groups: “loss of ≥ 4 kg (≤ −4 kg)”, “loss of ≥ 2 kg to loss of < 4 kg (> −4 to ≤  −2 kg)”, “loss of < 2 kg to gain of ≤ 2 kg (> −2 to ≤ 2 kg)”, “gain of > 2 kg to gain of ≤ 4 kg (> 2 to ≤ 4 kg)”, and “gain of > 4 kg (> 4 kg)”. Waist circumference change was also divided into five groups: “loss of ≥ 6 cm (≤ −6 cm)”, “loss of ≥ 3 cm to loss of < 6 cm (> −6 to ≤  −3 cm)”, “loss of < 3 cm to gain of ≤ 3 cm (> −3 to ≤ 3 cm)”, “gain of > 3 cm to gain of ≤ 6 cm (> 3 to ≤ 6 cm)”, and “gain of > 6 cm (> 6 cm)”.

All participants were asked to fast overnight before blood sample collection. Blood samples were transported to Beijing and stored at − 80℃ in the Chinese Center for Disease Control and Prevention. Bioassay was carried out at the laboratory of Capital Medical University. Fasting plasma glucose and total cholesterol were measured using enzymatic colormetric test. SUA, glycated hemoglobin, C-reactive protein, blood urea nitrogen, and serum creatinine were measured using UA Plus method, boronate affinity high-performance liquid chromatography, immunoturbidimetric assay, enzymatic ultraviolet method with urease, rate-blanked and compensated Jaffe creatinine method, respectively. Hyperuricemia was defined as a SUA value of ≥ 7 mg/dL for men, and a value of ≥ 6 mg/dL for women [25].

### Statistical analysis

Data analysis was performed using STATA software version 15.1 (StataCorp., T.X., USA). Data were described as mean (SD) or median (IQR) for continuous variables, frequency (percentage) for categorical variables. The normality of data was verified by Kolmogorov–Smirnov test. The between-group comparison was conducted using one-way analysis of variance (ANOVA) or Wilcoxon rank-sum test for continuous variables, and Pearson *χ*^2^ test was used for categorical variables. Adjusted odds ratio (OR) and corresponding 95% confidential interval (CI) were calculated using multivariate logistic regression to estimate the associations between predictor variables and hyperuricemia. Test for linear trend was performed by treating ordinal categorical variables as continuous variables in the logistic regression model. Area under the curve (AUC) was calculated through the receiver operating characteristic (ROC) curve analysis to compare the predictive power of different adiposity indicators for hyperuricemia. A *P* value of < 0.05 indicated a significant difference.

To further evaluate the joint effects of baseline BMI and weight change, baseline BMI and waist circumference change on risks of hyperuricemia, baseline BMI was recategorized into “ < 24 kg/m^2^”, “24–27.9 kg/m^2^”, and “≥ 28 kg/m^2^”. Weight change was recategorized into “loss of ≥ 4 kg (≤ −4 kg)”, “loss of < 4 kg to gain of ≤ 4 kg (> −4 to ≤ 4 kg)”, and “gain of > 4 kg (> 4 kg)”. Similarly, waist circumference change was recategorized into “loss of ≥ 6 cm (≤ −6 cm)”, “loss of < 6 cm to gain of ≤ 6 cm (> −6 to ≤ 6 cm)”, and “gain of > 6 cm (> 6 cm)”. Adjusted OR for incident hyperuricemia was also calculated under different combined statuses of baseline BMI/waist circumference and weight change or waist circumference change. Participants with baseline BMI of < 24 kg/m^2^ and weight change of > −4 to ≤ 4 kg, participants with baseline BMI of < 24 kg/m^2^ and waist circumference change of > −6 to ≤ 6 cm, participants with baseline waist circumference of < 90 cm (men)/85 cm (women) and weight change of > −4 to ≤ 4 kg, participants with baseline waist circumference of < 90 cm (men)/85 cm (women) and waist circumference change of > −6 to ≤ 6 cm were selected as the reference groups, respectively.

## Results

**Fig. 1:**
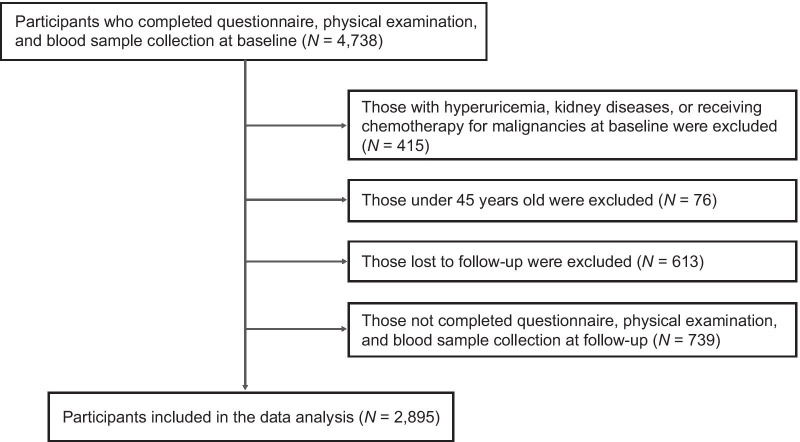
Flow chart for selection of study population

Figure 1 shows the flow chart for study population, and a total of 2895 participants were enrolled in the current study. The mean baseline age of participants was 59.08 years old, with males accounting for 49.36%. Of 2895 participants, 293 (10.12%) developed incident hyperuricemia. Individuals who developed hyperuricemia were more likely to be older, male, current drinkers; were more likely to have hypertension, dyslipidemia, and stroke; were more likely to have greater BMI, waist circumference, WHtR; and were more likely to have a higher concentration of SUA, total cholesterol, C-reactive protein, and serum creatinine at baseline (Table 1).

**Table 1:** Baseline characteristics of participants stratified by incident hyperuricemia status

Variables	Overall	Hyperuricemia		*P* value
		Yes	No	
Overall, *n* (%)	2895 (100.00)	293 (10.12)	2602 (89.88)	–
Age, year, mean (SD)	59.08 ± 8.94	60.58 ± 9.33	58.92 ± 8.88	*0.003*
Gender, *n* (%)				
Male	1429 (49.36)	164 (55.97)	1265 (48.62)	*0.017*
Female	1466 (50.64)	129 (44.03)	1337 (51.38)	
Residence, *n* (%)				
Rural	2453 (84.73)	241 (82.25)	2212 (85.01)	0.213
Urban	442 (15.27)	52 (17.75)	390 (14.99)	
Marital status, *n* (%)				
Not married	502 (17.34)	54 (18.43)	448 (17.22)	0.603
Currently married	2393 (82.66)	239 (81.57)	2154 (82.78)	
Education, *n* (%)				
Illiterate	689 (23.80)	60 (20.48)	629 (24.17)	0.305
Primary school and below	1291 (44.59)	132 (45.05)	1159 (44.54)	
Middle school and above	915 (31.61)	101 (34.47)	814 (31.28)	
Smoking history, *n* (%)				
No	1705 (58.89)	171 (58.36)	1534 (58.95)	0.845
Yes	1190 (41.11)	122 (41.64)	1068 (41.05)	
Drinking status, *n* (%)				
No	1931 (66.70)	177 (60.41)	1754 (67.41)	*0.016*
Yes	964 (33.30)	116 (39.59)	848 (32.59)	
BMI, kg/m^2^, mean (SD)	23.54 ± 3.62	24.82 ± 3.68	23.40 ± 3.58	< *0.001*
Waist circumference, cm, mean (SD)	85.48 ± 9.88	89.30 ± 9.62	85.05 ± 9.82	< *0.001*
WHtR, cm/cm, mean (SD)	0.54 ± 0.06	0.56 ± 0.06	0.54 ± 0.06	< *0.001*
Hypertension, *n* (%)				
No	2191 (75.68)	186 (63.48)	2005 (77.06)	< *0.001*
Yes	704 (24.32)	107 (36.52)	597 (22.94)	
Dyslipidemia, *n* (%)				
No	2621 (90.54)	249 (84.98)	2372 (91.16)	*0.001*
Yes	274 (9.46)	44 (15.02)	230 (8.84)	
Diabetes mellitus, *n* (%)				
No	2745 (94.82)	271 (92.49)	2474 (95.08)	0.058
Yes	150 (5.18)	22 (7.51)	128 (4.92)	
Stroke, *n* (%)				
No	2839 (98.07)	281 (95.90)	2558 (98.31)	*0.005*
Yes	56 (1.93)	12 (4.10)	44 (1.69)	
Depression, *n* (%)				
No	1796 (62.04)	194 (66.21)	1602 (61.57)	0.120
Yes	1099 (37.96)	99 (33.79)	1000 (38.43)	
SUA, mg/dL, mean (SD)	4.30 ± 1.04	5.29 ± 0.94	4.19 ± 0.99	< *0.001*
Fasting plasma glucose, mg/dL, mean (SD)	109.26 ± 32.25	111.71 ± 34.50	108.99 ± 31.99	0.171
Glycated hemoglobin, %, mean (SD)	5.25 ± 0.75	5.28 ± 0.79	5.25 ± 0.75	0.466
Total cholesterol, mg/dL, mean (SD)	192.53 ± 36.34	198.63 ± 33.31	191.84 ± 36.61	*0.002*
C-reactive protein, mg/L, median (IQR)	0.98 (0.54–2.05)	1.31 (0.70–2.67)	0.95 (0.52–1.95)	< *0.001*
Blood urea nitrogen, mg/dL, mean (SD)	15.58 ± 4.29	15.83 ± 3.69	15.56 ± 4.36	0.300
Serum creatinine, mg/dL, mean (SD)	0.77 ± 0.17	0.84 ± 0.18	0.76 ± 0.17	< *0.001*

*BMI* body mass index, *IQR* interquartile range, *SD* standard deviation, *SUA* serum uric acid; WHtR, waist-height ratio

Values in italics are statistically significant (*P* < 0.05)

**Fig. 2:**
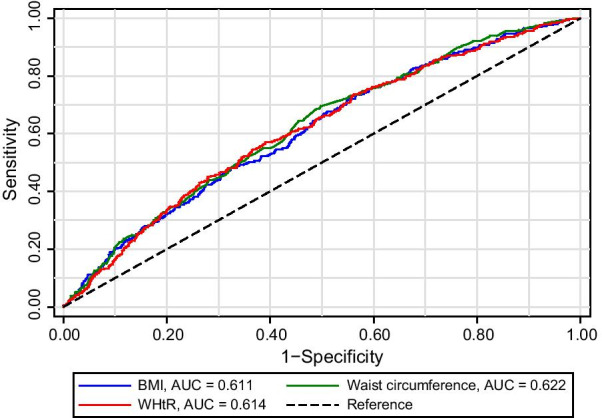
ROC curves for incident hyperuricemia comparing BMI, waist circumference, and WHtR

Multivariate logistic regression (Model 3) revealed that participants with obesity (BMI of ≥ 28 kg/m^2^) were at higher risk of hyperuricemia (OR 1.76; 95% CI 1.18–2.64) compared to those with BMI of 18.5–23.9 kg/m^2^, whereas there was no significant association between BMI and hyperuricemia risk in individuals with underweight (BMI of < 18.5 kg/m^2^) or overweight (BMI of 24–27.9 kg/m^2^). A linear trend toward higher risks of hyperuricemia as BMI increased was detected (*P* for trend = 0.022, Table 2). When waist circumference was applied, a significantly increased risk of hyperuricemia was observed in the third and fourth quartile compared to the first quartile (OR 1.84; 95% CI 1.20–2.83 and OR 2.12; 95% CI 1.38–3.27, respectively) (*P* for trend < 0.001, Table 2). In comparison with the first quartile of WHtR, the adjusted OR (Model 3) for incident hyperuricemia in the second, third and fourth quartile were 1.51 (95% CI 0.97–2.36), 1.61 (95% CI 1.03–2.51), and 2.75 (95% CI 1.75–4.31), respectively (*P* for trend < 0.001, Table 2). Compared to participants without abdominal obesity [waist circumference of < 90 cm (men)/85 cm (women)] or with low WHtR (< 0.5), individuals with abdominal obesity or high WHtR had a 1.57-fold (95% CI 1.17–2.10) or a 1.78-fold (95% CI 1.24–2.55) increased risk of hyperuricemia (Table 2). The adjusted OR (Model 3) for incident hyperuricemia per SD increase in BMI, waist circumference, and WHtR were 1.36 (95% CI 1.17–1.57), 1.34 (95% CI 1.17–1.53), and 1.42 (95% CI 1.22–1.65), respectively. In addition, ROC curve analysis showed a slightly higher AUC value for waist circumference (0.622) than BMI (0.611) and WHtR (0.614), but the intergroup difference was not statistically significant (*P* = 0.447, Fig. 2), demonstrating the comparable predictive ability of these indicators for incident hyperuricemia.

**Table 2:** Odds ratios of incident hyperuricemia associated with BMI, waist circumference, and WHtR in middle-aged and older Chinese population

Variables	Overall, *n*	Case, *n* (%)	OR (95% CI)		
			Model 1	Model 2	Model 3
BMI, kg/m^2^					
< 18.5	173	7 (4.05)	*0.41 (0.19–0.91)*	*0.44 (0.20–0.97)*	0.49 (0.21–1.12)
18.5–23.9	1542	133 (8.63)	1.00	1.00	1.00
24–27.9	831	93 (11.19)	*1.49 (1.11–1.99)*	1.32 (0.98–1.77)	1.19 (0.86–1.64)
≥ 28	349	60 (17.19)	*2.71 (1.91–3.85)*	*2.20 (1.52–3.18)*	*1.76 (1.18–2.64)*
*P* for trend			< *0.001*	< *0.001*	*0.022*
BMI, per SD increase (3.62 kg/m^2^)	2895	293 (10.12)	*1.60 (1.42–1.82)*	*1.49 (1.31–1.70)*	*1.36 (1.17–1.57)*
Waist circumference (quartiles), cm					
First (men, < 77.5; women, < 79.1)	721	38 (5.27)	1.00	1.00	1.00
Second (men, 77.5–83.9; women, 79.1–85.9)	698	54 (7.74)	1.47 (0.96–2.27)	1.39 (0.90–2.14)	1.38 (0.87–2.18)
Third (men, 84–90.9; women, 86–93.09)	744	88 (11.83)	*2.40 (1.61–3.58)*	*2.20 (1.47–3.28)*	*1.84 (1.20–2.83)*
Fourth (men, ≥ 91; women, ≥ 93.1)	732	113 (15.44)	*3.24 (2.20–4.78)*	*2.67 (1.78–4.00)*	*2.12 (1.38–3.27)*
*P* for trend			< *0.001*	< *0.001*	< *0.001*
Waist circumference, cm					
< 90 (men) or < 85 (women)	1665	127 (7.63)	1.00	1.00	1.00
≥ 90 (men) or ≥ 85 (women)	1230	166 (13.50)	*2.15 (1.66–2.78)*	*1.86 (1.42–2.43)*	*1.57 (1.17–2.10)*
Waist circumference, per SD increase (9.88 cm)	2895	293 (10.12)	*1.55 (1.37–1.76)*	*1.45 (1.27–1.65)*	*1.34 (1.17–1.53)*
WHtR (quartiles)					
First (< 0.493)	723	39 (5.39)	1.00	1.00	1.00
Second (0.493–0.534)	724	67 (9.25)	*1.88 (1.24–2.84)*	*1.79 (1.18–2.71)*	1.51 (0.97–2.36)
Third (0.535–0.584)	724	74 (10.22)	*2.26 (1.50–3.41)*	*2.02 (1.33–3.06)*	*1.61 (1.03–2.51)*
Fourth (≥ 0.585)	724	113 (15.61)	*4.35 (2.89–6.55)*	*3.65 (2.40–5.56)*	*2.75 (1.75–4.31)*
*P* for trend			< *0.001*	< *0.001*	< *0.001*
WHtR					
< 0.5	823	48 (5.83)	1.00	1.00	1.00
≥ 0.5	2072	245 (11.82)	*2.41 (1.73–3.36)*	*2.13 (1.52–2.98)*	*1.78 (1.24–2.55)*
WHtR, per SD increase (0.065)	2895	293 (10.12)	*1.66 (1.45–1.89)*	*1.55 (1.35–1.78)*	*1.42 (1.22–1.65)*

Model 1: Adjusted for age, gender, residence, marriage, education, smoking history, and drinking status

Model 2: Adjusted for all variables in Model 1 plus hypertension, dyslipidemia, diabetes mellitus, stroke, and depression

Model 3: Adjusted for all variables in Model 2 plus fasting plasma glucose, glycated hemoglobin, total cholesterol, C-reactive protein, blood urea nitrogen, creatinine, and baseline SUA

*BMI* body mass index, *CI* confidence interval, *OR* odds ratio, *SD* standard deviation, *SUA* serum uric acid, *WHtR* waist-height ratio

Values in italics are statistically significant (*P* < 0.05)

Compared to those with stable weight during follow-up (> −2 to ≤ 2 kg), there was a 56% or 37% lower risk of hyperuricemia (Model 3) in participants with weight loss of ≥ 4 kg or weight loss of ≥ 2 to < 4 kg. Besides, a 1.62-fold increased OR in participants with weight gain of > 4 kg was found (*P* for trend < 0.001). Similarly, participants with waist circumference loss of ≥ 6 cm had a reduced risk of hyperuricemia in comparison with those with waist circumference loss of < 3 cm to gain of ≤ 3 cm (OR 0.45; 95% CI 0.27–0.73; *P* for trend = 0.001). The adjusted OR (Model 3) for incident hyperuricemia per SD increase in weight change and waist circumference change was 1.37 (95%CI 1.21–1.55) and 1.27 (95% CI 1.11–1.46), respectively (Table 3). Further, we also explored the impact of changes in obesity status on hyperuricemia risk. Compared to those without obesity both at baseline and at follow-up, individuals with incident or persistent general obesity had a 3.04-fold (95% CI 1.66–5.59) or a 2.22-fold (95% CI 1.49–3.31) higher risk of hyperuricemia. Similarly, incident or persistent abdominal obesity was associated with higher risks of hyperuricemia, with an adjusted OR of 1.88 (95% CI 1.18–2.98) or 2.14 (95% CI 1.54–2.97) (Table 4).

**Table 3:** Odds ratios of incident hyperuricemia associated with changes in weight and waist circumference in middle-aged and older Chinese population

Variables	Overall, *n*	Case, *n* (%)	OR (95% CI)		
			Model 1	Model 2	Model 3
Weight change, kg					
Loss of ≥ 4	322	21 (6.52)	*0.56 (0.35–0.91)*	*0.50 (0.31–0.82)*	*0.44 (0.26–0.74)*
Loss of ≥ 2 to loss of < 4	412	33 (8.01)	0.71 (0.48–1.06)	*0.67 (0.45–1.00)*	*0.63 (0.41–0.98)*
Loss of < 2 to gain of ≤ 2	1364	146 (10.70)	1.00	1.00	1.00
Gain of > 2 to gain of ≤ 4	415	43 (10.36)	1.00 (0.69–1.43)	0.98 (0.68–1.41)	0.96 (0.64–1.43)
Gain of > 4	382	50 (13.09)	1.34 (0.95–1.90)	1.39 (0.98–1.97)	*1.62 (1.10–2.37)*
*P* for trend			*0.001*	< *0.001*	< *0.001*
Weight change, per SD increase (4.25 kg)	2895	293 (10.12)	*1.24 (1.11–1.39)*	*1.27 (1.13–1.42)*	*1.37 (1.21–1.55)*
Waist circumference change, cm					
Loss of ≥ 6	361	23 (6.37)	*0.51 (0.32–0.81)*	*0.51 (0.32–0.80)*	*0.45 (0.27–0.73)*
Loss of ≥ 3 to loss of < 6	315	28 (8.89)	0.73 (0.48–1.12)	0.73 (0.47–1.12)	0.65 (0.41–1.04)
Loss of < 3 to gain of ≤ 3	1219	141 (11.57)	1.00	1.00	1.00
Gain of > 3 to gain of ≤ 6	501	38 (7.58)	*0.65 (0.45–0.95)*	*0.65 (0.45–0.95)*	0.66 (0.44–1.03)
Gain of > 6	499	63 (12.63)	1.15 (0.83–1.58)	1.19 (0.86–1.64)	1.24 (0.87–1.76)
*P* for trend			*0.011*	*0.007*	*0.001*
Waist circumference change, per SD increase (6.14 cm)	2895	293 (10.12)	*1.20 (1.06–1.36)*	*1.21 (1.07–1.38)*	*1.27 (1.11–1.46)*

Model 1: Adjusted for age, gender, residence, marriage, education, smoking history, and drinking status

Model 2: Adjusted for all variables in Model 1 plus hypertension, dyslipidemia, diabetes mellitus, stroke, and depression

Model 3: Adjusted for all variables in Model 2 plus fasting plasma glucose, glycated hemoglobin, total cholesterol, C-reactive protein, blood urea nitrogen, creatinine, and baseline SUA

*CI* confidence interval, *OR* odds ratio, *SD* standard deviation, *SUA* serum uric acid

Values in italics are statistically significant (*P* < 0.05)

**Table 4:** Odds ratios of incident hyperuricemia stratified by obesity status at baseline and follow-up

Obesity at baseline	Obesity at follow up	Overall, *n*	Case, *n* (%)	OR (95% CI)		
				Model 1	Model 2	Model 3
General obesity^†^						
No^(a)^	No	2451	215 (8.77)	1.00	1.00	1.00
No^(b)^	Yes	95	18 (18.95)	*3.17 (1.83–5.49)*	*3.05 (1.76–5.31)*	*3.04 (1.66–5.59)*
Yes^(c)^	No	85	8 (9.41)	1.23 (0.58–2.62)	1.04 (0.49–2.23)	0.72 (0.32–1.63)
Yes^(d)^	Yes	264	52 (19.70)	*3.05 (2.16–4.32)*	*2.51 (1.75–3.62)*	*2.22 (1.49–3.31)*
Abdominal obesity^††^						
No^(a)^	No	1346	94 (6.98)	1.00	1.00	1.00
No^(b)^	Yes	319	33 (10.34)	*1.83 (1.20–2.81)*	*1.75 (1.14–2.69)*	*1.88 (1.18–2.98)*
Yes^(c)^	No	191	10 (5.24)	0.82 (0.42–1.61)	0.75 (0.38–1.49)	0.60 (0.29–1.23)
Yes^(d)^	Yes	1039	156 (15.01)	*2.87 (2.15–3.83)*	*2.48 (1.83–3.35)*	*2.14 (1.54–2.97)*

Model 1: Adjusted for age, gender, residence, marriage, education, smoking history, and drinking status

Model 2: Adjusted for all variables in Model 1 plus hypertension, dyslipidemia, diabetes mellitus, stroke, and depression

Model 3: Adjusted for all variables in Model 2 plus fasting plasma glucose, glycated hemoglobin, total cholesterol, C-reactive protein, blood urea nitrogen, creatinine, and baseline SUA

*BMI* body mass index, *CI* confidence interval, *OR* odds ratio, *SUA* serum uric acid

Change in obesity status is defined as (a) no obesity, (b) incident obesity, (c) remittent obesity, and (d) persistent obesity

^†^General obesity is determined using BMI; ^††^Abdominal obesity is determined using waist circumference

Values in italics are statistically significant (*P* < 0.05)

**Fig. 3:**
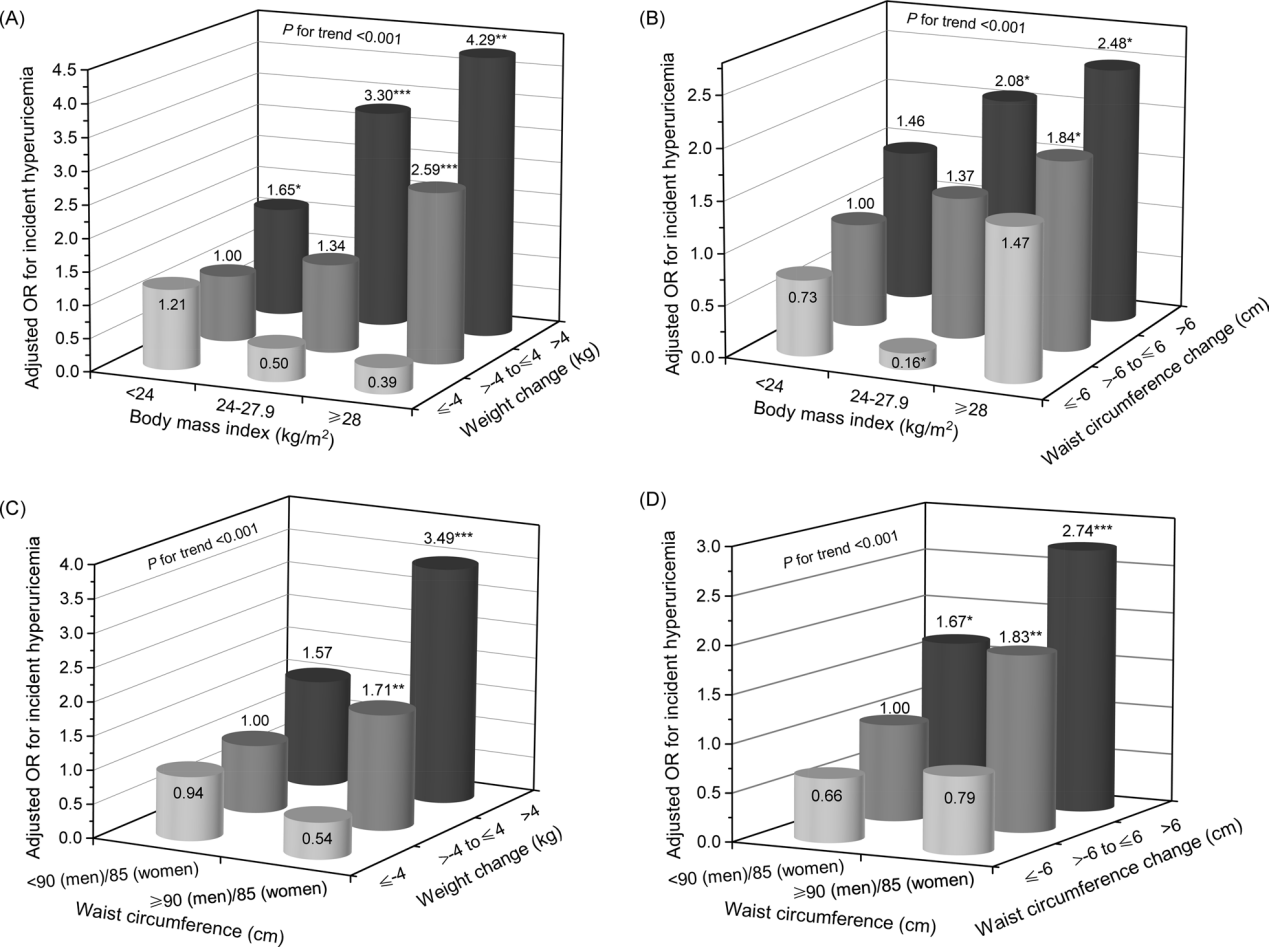
Adjusted OR for incident hyperuricemia was calculated, with a combined status of **a** BMI of < 24 kg/m^2^ and weight change of > −4 to ≤ 4 kg, **b** BMI of < 24 kg/m^2^ and waist circumference change of > −6 to ≤ 6 cm, **c** non-abdominal obesity [< 90 cm (men)/85 cm (women)] and weight change of > −4 to ≤ 4 kg, and **d** non-abdominal obesity and waist circumference change of > −6 to ≤ 6 cm as the reference groups, respectively. *P < 0.05, **P < 0.01, ***P < 0.001

In addition, this study explored the joint effects of baseline BMI and changes in weight or waist circumference, baseline waist circumference and changes in weight or waist circumference on risks of hyperuricemia. Participants with obesity and had weight gain of > 4 kg exhibited a 4.29-fold (95% CI 1.66–11.09) increased risk of hyperuricemia in comparison with those who had BMI of < 24 kg/m^2^ and had stable weight (> −4 to ≤ 4 kg). Participants with obesity and had waist circumference gain of > 6 cm exhibited a 2.48-fold (95% CI 1.11–5.55) increased risk of hyperuricemia in comparison with those who had BMI of < 24 kg/m^2^ and had stable waist circumference (> −6 to ≤ 6 cm). Similarly, we found a significant association of increased hyperuricemia risk with a combined status of abdominal obesity [waist circumference of ≥ 90 cm (men)/85 cm (women)] and weight gain of > 4 kg compared to those without abdominal obesity and had stable weight (OR 3.49; 95% CI 2.06–5.91). Also, there was a significant association of increased hyperuricemia risk with a combined status of abdominal obesity and waist circumference gain of > 6 cm compared to those without abdominal obesity and had stable waist circumference (OR 2.74; 95% CI 1.57–4.77) (Fig. 3).

## Discussion

The present study demonstrated that indicators of both general obesity and abdominal obesity were related to a higher risk of hyperuricemia in middle-aged and older Chinese population, and loss of weight or waist circumference may help reduce the risk. Also, both development of obesity and persistent obesity were found to be risk factors for incident hyperuricemia. BMI, waist circumference, and WHtR exhibited comparable predictive power for incident hyperuricemia. Additionally, we found for the first time a significant joint association of hyperuricemia risk with a combined status of baseline BMI and weight change or waist circumference change, as well as the combined status of baseline waist circumference and weight change or waist circumference change.

The present study showed that the cumulative incidence of hyperuricemia was 10.12% in middle-aged and older Chinese population, with a follow-up duration of 4 years. The hyperuricemia incidence is comparable with that reported in a study by Dong et al. (10.24%) [26], but less than another finding (20.6%) [25], which is possibly due to different durations of follow-up. Previous studies have shown a positive relationship between obesity and SUA [27–29], which is in line with our findings. In this study, we found that both general obesity and abdominal obesity were associated with a higher risk of hyperuricemia, and this association maintained even further adjusted for baseline SUA. ROC curve analysis showed a slightly greater but non-significant AUC value for waist circumference compared with BMI and WHtR, indicating the equal predictive value of these adiposity indices for incident hyperuricemia. Furthermore, we did not observe a notable difference in the adjusted OR (Model 3) per SD increase in BMI, waist circumference, and WHtR, which were 1.36, 1.34, and 1.42, respectively. Similar to our findings, a study [30] involving 5685 Chinese individuals aged ≥ 60 years reported that there was no significant difference in the AUC of BMI and waist circumference for predicting elevated SUA in both males and females. However, another cross-sectional study [31] conducted in China suggested that WHtR was a better predictor of hyperuricemia than BMI and waist circumference. The discrepancy may be partially explained by two aspects. First, abdominal fat accumulation leads to weight gain, subsequently contributing to an increase in BMI. Through post hoc analysis, we found that a huge overlap existed between participants with BMI ≥ 28 kg/m^2^ and those with abdominal obesity in this study. Second, it is not feasible to delineate the temporal relationship between risks of hyperuricemia and these predictors in previous studies for their cross-sectional study design. Higher SUA level has been shown to induce abdominal obesity [32], this might account for a stronger association between WHtR and hyperuricemia reported in previous studies.

Previous studies have mainly focused on the relationship between changes in weight or waist circumference and changes in SUA [17, 33, 34], but whether such changes can indeed lead to the development of hyperuricemia remains inconclusive. The current study showed that loss of weight or waist circumference was favorable in reducing risks of hyperuricemia, and individuals with a gain in weight or waist circumference showed a higher propensity to develop hyperuricemia. In line with our results, a study with a ten-year follow-up period analyzed the data of 2611 participants aged 17–35 years from the Coronary Artery Risk Development in Young Adults (CARDIA) Study, and found that a decrease in BMI and waist circumference was associated with a lower SUA level after multivariate adjustments [34]. Similar findings have also been reported in another study by Heyden et al. and colleagues [33]. In our study, the results indicated that participants who developed obesity during follow-up were more inclined to suffer hyperuricemia, and persistent obesity also facilitated the development of hyperuricemia, further supporting the above findings. Also, a trend toward a lower risk of hyperuricemia for those with remittent obesity was observed, but this association was not statistically significant. The findings in this group should be verified by future research given the limited number of hyperuricemia cases (general obesity, *n* = 8; abdominal obesity, *n* = 10). Joint association analysis revealed that weight maintenance and waist circumference maintenance among individuals with general obesity were not enough to reduce risks of hyperuricemia, and a gain in weight or waist circumference may significantly increase the risk. Similar findings were also detected in subjects with abdominal obesity.

Decreased excretion and increased production of SUA may explain the high risk of incident hyperuricemia in obese individuals [35]. SUA concentration is highly dependent on renal excretion [34], while obesity leads to an increase in glucose concentration and blood pressure, risk factors for renal functions [36, 37]. In addition, insulin resistance intrigued by higher BMI can cause damage to urinary tract function, leading to a decrease in excretion of uric acid [9]. Additionally, visceral fat promotes the synthesis of very low-density lipoprotein and ribose-5-phosphate to phosphoribosyl pyrophosphate, which contribute to overproduction of uric acid [38]. Furthermore, greater catalytic activity of xanthine oxidoreductase is observed in obese individuals, facilitating the synthesis of uric acid [39, 40]. However, the underlying mechanisms have not been fully understood and warrant further explorations.

The present study has several strengths. First, this study was conducted using the data from the CHARLS, a population-based nationwide survey, ensuring sufficient representativeness of our study population. Second, we compared the predictive value of BMI, waist circumference, and WHtR using a longitudinal data set, and elucidated the relationship of changes in obesity status (general obesity and abdominal obesity) with risks of hyperuricemia. Third, we explored the joint associations of baseline adiposity indices and body adiposity change with hyperuricemia risk for the first time. However, this study also has some limitations. First, although our analysis made adjustments for a variety of confounding variables, the influence of other variables, like intake of dairy, meat, microelements, and use of allopurinol [41–43], cannot be ruled out since these data were not collected in the survey. Second, the participants in this study are Chinese population aged ≥ 45 years, thus it should be cautious when interpreting our findings in younger population and other ethnic individuals. Third, the follow-up duration is 4 years, impeding a further exploration of long-term associations between predictor variables and risks of hyperuricemia.

## Conclusion

The present study demonstrates that BMI, waist circumference, and WHtR are comparable predictors of hyperuricemia in middle-aged and older Chinese population, and weight loss and waist circumference reduction are favorable in reducing the risk. Avoiding excessive gain in weight and waist circumference is an effective approach to prevent hyperuricemia, especially for individuals with overweight or obesity.

## Data Availability

The datasets generated and/or analyzed during the current study are publicly available on the following website: http://charls.pku.edu.cn/en.

## Abbreviations

AUC: Area under the curve
BMI: Body mass index
CES-D: The Center for Epidemiologic Studies Depression Scale
CHARLS: The China Health and Retirement Longitudinal Study
CI: Confidential interval
IQR: Interquartile range
OR: Odds ratio
ROC: Receiver operating characteristic
SD: Standard deviation
SUA: Serum uric acid
WHtR: Waist-height ratio
